# Supplementary material for: Variability by region and method in human brain sodium concentrations estimated by 23Na magnetic resonance imaging: a meta-analysis
Source: Sci Rep. 2023 Feb 24;13:3222. doi: 10.1038/s41598-023-30363-y (PMC9957999; doi:10.1038/s41598-023-30363-y)
Supplement: Supplementary file 1 — Supplementary Figure 1. [file 41598_2023_30363_MOESM1_ESM.docx]

**
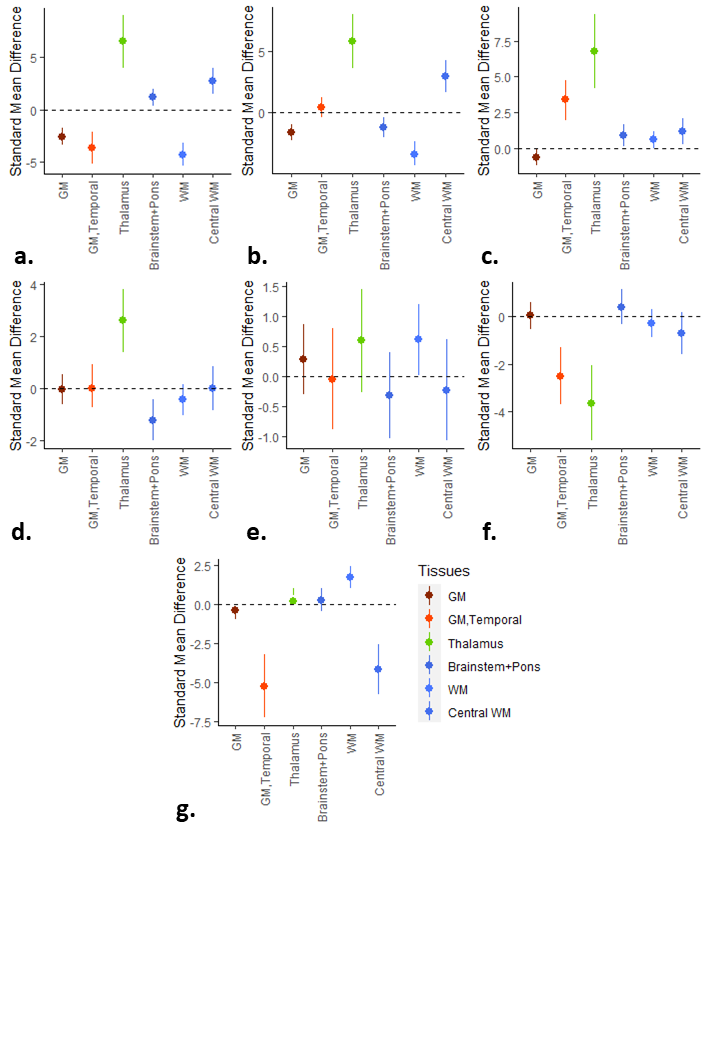
**

**Supplementary Figure 1: Scatterplots of standardized mean differences (Hedges’ g) in regional heterogeneity (tau) between models with and without methodological moderators.** a) Sequence, b) Comparison group, c) Calibration method, d) Voxel volume, e) Field Strength, f) TR, g) TE. Error bars correspond to 95% confidence intervals. Images created in R^64–66^
